# An Assessment of the Catalytic and Adsorptive Performances of Cellulose Acetate-Based Composite Membranes for Oil/Water Emulsion Separation

**DOI:** 10.3390/polym16223108

**Published:** 2024-11-05

**Authors:** Mahendran Gurusamy, Sangeetha Thangavel, Jakub Čespiva, Jiří Ryšavý, Wei-Mon Yan, Marek Jadlovec, Gangasalam Arthanareeswaran

**Affiliations:** 1 Membrane Research Laboratory, Department of Chemical Engineering, National Institute of Technology, Tiruchirappalli 620 015, Tamil Nadu, India; 2 Department of Energy and Refrigerating, Air-Conditioning Engineering, National Taipei University of Technology, Taipei 10608, Taiwan; 3 Research Center of Energy Conservation for New Generation of Residential, Commercial, and Industrial Sectors, National Taipei University of Technology, Taipei 10608, Taiwan; 4 Energy Research Centre, Centre for Energy and Environmental Technologies, VSB–Technical University of Ostrava, 17. Listopadu 2172/15, 70800 Ostrava-Poruba, Czech Republic; jiri.rysavy@vsb.cz; 5 Energy Department, Faculty of Mechanical Engineering, VSB–Technical University of Ostrava, 17. Listopadu 2172/15, 70800 Ostrava-Poruba, Czech Republic; marek.jadlovec@vsb.cz

**Keywords:** cellulose acetate (CA), polyvinylpyrrolidone (PVP), graphene oxide (GO), bentonite (B), titanium dioxide (TiO_2_), toluene, hexane, oil/water emulsion

## Abstract

Cellulose acetate (CA) mixed-matrix membranes incorporating polyvinylpyrrolidone (PVP), bentonite (B or Ben), graphene oxide (GO), and titanium dioxide (TiO_2_) were prepared by the phase inversion separation technique for oil/water separation. An investigation was performed where the mixed-matrix membrane was tested for the separation performance of hydrophilic and hydrophobic surface properties. An ultrafiltration experiment at the laboratory scale was used to test dead-end ultrafiltration models developed for the treatment performances of oily wastewater under dynamic full-scale operating conditions. Artificial oily wastewater solutions were prepared from hexane, toluene, and engine oil with Tween80 emulsions for oil removal treatment using composite membranes. The impacts of material hydrophilicity, weight loss, permeability, and pore size were investigated, and it was found that the oil retention of membranes with larger pore sizes enabled much more sophisticated water flux. The CA-GO-, CA-B-, and CA-TiO_2_-incorporated membranes achieved pure water flux (PWF) values of 45.19, 53.41, and 100.25 L/m^2^h, respectively. The performance of CA-TiO_2_ in oil/water emulsion rejection was assessed, and the rejection of engine oil/water, toluene/water, and hexane/water mixtures was determined to be 95.21%, 90.33%, and 92.4%, respectively. The CA-based mixed-matrix membrane portrayed better antifouling properties due to enhanced hydrophilicity and water molecules. The CA-TiO_2_-incorporated membrane possessed the potential to provide high separation efficiency for oily wastewater treatment. This study demonstrates the potential of fine-tuning membrane performances through material hybridization to achieve efficient wastewater treatment.

## 1. Introduction

The world is facing serious environmental problems, such as water shortages and pollution, due to oily wastewater industries [1,2]. Simultaneously, high concentrations of 100–1000 mg L^−1^ of oily wastewater are discharged into the environment from industrial operations [3]. Membrane separation technology is preferred for the treatment of oily wastewater as it demonstrates economic and environmentally friendly advantages such as low treatment costs and the reuse of water or oil in industries [4]. Membrane separation technology methods enhance the separation of small oil droplets from oily wastewater by pressure-driven membrane processes [5,6,7,8]. They are capable of removing oil droplets smaller than 9–10 μm [9,10]. Pressure is employed in ultrafiltration (UF) on the feed side, permeate, and retentate for the treatment of wastewater [11] at various operating conditions regarding concentration, pressure, UV radiation, acidity or alkalinity, and temperature [12,13]. Ultrafiltration membrane processes have several advantages over conventional oil/water separation strategies, including higher efficiency, greater selectivity, reduced environmental impact, lower maintenance requirements, better automation possibilities, and greater adaptability to varying feed conditions. However, there are fewer drawbacks of conventional oil/water separation strategies compared to other membrane processes [14]. UF methods are desired as they reject oil droplets and smaller emulsified droplets due to small membrane pores [15]. CA is generally highly adsorptive, durable, stable, biodegradable, and selective and has applications in the field of UF [16,17,18]. CA exhibits an affinity for hydrophilic materials and hydroxyl groups, and has film-forming and oleophobic properties compared with other polymeric membranes [19,20]. Membrane cleaning is essential, and if rarely performed, the deposition of small oil droplets on the membrane surface will result in devaluation and the low-separation-efficiency conditions of water flux. Ultrafiltration requires high transmembrane pressure and flux [21]. The nanoparticles of ZrO_2_ [22], TiO_2_, Al_2_O_3_ [23], SiO_2_ [24] (Ding et al., 2019), bentonite [25], and GO have been utilized in previous studies to enhance the hydrophilic characteristics of polymeric membranes. Surface coating, polymer blending, chemical treatment, and surface grafting techniques have been applied to reduce the fouling of incorporated membranes [26]. Surface modification improves the surface charge, smoothness, hydrophilicity, and high-molecular-weight polymers in the membrane [27]. The chemical treatment method is also performed for by-product recovery from waste, which thereby reduces the overall expenses of waste disposal [28]. Surface grafting techniques for polymers are challenging to scale up and transform for pilot plants [29,30]. The blending method is enforced for flat sheet membranes and is easily modified by hydrophilic additives such as polymers and nanoparticles [31]. The blending method [32,33,34] of hydrophilic additives is advantageous for solving membrane fouling problems.

The uniform dispersal of nanomaterials tends to modify their porous structure and porosity, thereby creating free channels for water transport in the membrane matrix. Nanomaterials such as graphene oxide (GO)/titanium dioxide (TiO_2,_) have been proven to enhance fouling resistance by increasing membrane hydrophilicity. Xu et al. [35] tested graphene oxide/TiO_2_ composite filtration membranes to enhance fouling resistance in water purification applications. Titanium dioxide (TiO_2_) and graphene oxide (GO) exhibit characteristics like good absorption and can remove oil content from water. Various research studies have reported that mixed-matrix membranes prepared by incorporating zeolite 4A into the polyimide of matrimid 5218 for the pervaporation dehydration of isopropanol were very advantageous, and metal/organic framework-71 (MOF-71), silicon dioxide (SiO_2_), activated carbon (AC), and halloysite nanotube (HNT)-incorporated PEIs were used as the nanofiller of membranes for oil/water emulsion treatment [36,37]. Carbon-based mixed-matrix membranes with hydrophilic carbon nanoparticles enhanced properties like membrane permeability, longevity, and antifouling capacity and proved to be a versatile material as an engineering nanomaterial for fabricating advanced mixed-matrix membranes. Antifouling or low-fouling membranes have been developed to restrict oil fouling problems and reduce permeate flux decay [11]. The fabrication of a hybrid membrane using the blending process has been considered a promising strategy that can have prominent impacts on scalability for industrial applications. Nanoparticle-blended membrane nanomaterials have numerous advantages like ease of preparation, repeatability, and commercial sustainability.

Kusworo et al. [38] subjected oil field wastewater to water treatment using the integrated activated carbon–bentonite adsorbent and double-stage membrane process. Analogous studies were also performed using GO and TiO_2_ composite ultrafiltration membranes, which yielded encouraging findings for applications involving wastewater treatment [39]. Though these nanoparticles, polymers, and clays have been incorporated into CA ultrafiltration membranes for the treatment of oil and water, their assessment, interactions, and performance parameters have not yet been investigated in detail as far as the research knowledge of the authors is concerned. Therefore, we conducted a research investigation to explore and assess the catalytic and adsorptive performance of CA membranes for the effective removal of oils and separation of water-in-oil emulsions, which is considered a novel endeavor.

In the present research study, composite mixed-matrix membranes were designed using TiO_2_, GO, and bentonite in CA with PVP polymers following the phase inversion method. A hybrid membrane study for oil/water separation with hydrophilic and hydrophobic surface properties has also been reported. The incorporated mixed-matrix membrane modules were employed for oil/water separation by preparing hexane, toluene, and engine oil [40]. Moreover, CA-TiO_2_-incorporated membranes exhibited improved antifouling performance, hydrophilicity, reduced membrane fouling, and potential applications. Consequently, our research team conducted a study to compare the efficacy of various membranes for ultrafiltration and oil/water treatment. These membranes incorporated nanomaterials, polymers, and clay CA. Engine oil/water, hexane/water, and toluene/water emulsions were used as foulants, and the impacts of various nanomaterials in the casting solution in terms of membrane hydrophilicity, permeate flux, morphology, and antifouling properties were estimated. The experimental outcomes of this study will pave the way for the real-time implementation of these innovative membranes and eventually to their successful commercialization.

## 2. Experimental Methodology

### 2.1. Materials Acquired

The materials (CA, polyvinylpyrrolidone (PVP)) required for this experimental study were procured from Sigma-Aldrich, St. Louis, MO, USA. The nanoparticles such as graphene oxide (GO), titanium dioxide (TiO_2_), and nanoclay of bentonite (B or Ben), solvents such as N-Methyl-2-Pyrrolidone (NMP), toluene, and hexane were obtained from Sisco Research Laboratories Pvt. Ltd., Mumbai, India, and Tween-80 emulsifier was procured from Merck Life science Pvt. Ltd., Bangalore, India. Engine oil was purchased from the Annai Engine Care, Kattur, Tiruchirappalli, India. Distilled water was used for washing and solution preparation.

### 2.2. Preparation of Incorporated Mixed-Matrix Membrane

Fabrication of CA blended with PVP [41], GO [42], Bentonite [43], and TiO_2_ [39] mixed-matrix membranes was performed. The casting solution was prepared by mixing the solvent with CA in a round-bottom flask, followed by the addition of PVP, bentonite, TiO_2_, and GO nanoparticles and constant stirring for 4 h at 30 °C to obtain a homogenous solution. The phase inversion method was utilized for the preparation of membranes by the addition of nanoparticles in the ratio of 0.5 wt% with 17.0 wt% of CA in the presence of solvent NMP 82.5 wt%.

### 2.3. Oil/Water Emulsion Formulation

The protocol followed for the preparation of the stabilized oil-in-water emulsions was in accordance with Pagidi et al. [44]. Initially, 10 mg of Tween80 was mixed with 990 mL water. Subsequently, 10 mL of engine oil, 10 mL of n-Hexane, and 10 mL of toluene were added in separate beakers and were all covered with aluminum foils. Oil-in-water emulsions were ultrasonicated and continuously stirred with a high-shear homogenizer for 24 h at 30 °C to obtain a stable mixture of water emulsions of hexane, toluene, and engine oil.

### 2.4. Characterization

Scanning Electron Microscopy (SEM, S–3400 N, Hitachi, Chiyoda City, Japan) was used to investigate the top surface morphology of membranes, whereas the cross-sectional morphology and EDAX of fabricated incorporated membranes were evaluated by Field Emission Scanning Electron Microscopy (FESEM, FEI 400FEG, Hillsboro, OR, USA). Fourier Transform Infrared Spectroscopy (FTIR) was performed on a Nicolet iS50 system (Thermo Fisher Scientific, Bangalore, India) and the fabricated membranes’ contact angles (CAs) were verified by an OSA60 (LAUDA Scientific India Ltd., Mumbai, India). The stability of membranes was investigated using a tensile testing machine.

### 2.5. Membrane Performances

The bulk porosity (*ε*) of the membrane was calculated using a piece of wet membrane (4 cm × 4 cm) which was then dried at 60 °C to a constant weight. The equation is

(1) $$\varepsilon =\frac{Mw-Md}{S\times l\times \rho }\times 100$$

*Mw* and *Md* (g) are the wet and dry membrane mass, respectively, where *S* (cm^2^) is the area of the membrane, *l* (cm) is the thickness of the membrane, and *ρ* (g/cm^3^) is the density of pure water.

The following equations were used to calculate the Swelling Ratio, %. It is indirectly related to the porosity and degree of hydrophilicity of the membranes:

(2) $$\mathrm{Swelling}\;\mathrm{Ratio}\;\%=\frac{Ww-Wd}{Wd}\times 100$$

where *Ww* is the wet sample weight (g) and *Wd* is the dry sample weight (g).

The emulsion was then poured onto a filtration device equipped with a membrane. The emulsion separation was carried out under constant pressure of 1 bar. The separation efficiency was calculated as follows:

(3) $$R=\frac{Cf-Cp}{Cf}\times 100$$

*Cp* is the oil concentration in the permeate, and C_f_ is the oil concentration in the feed measured by a UV spectrophotometer.

The flux was calculated as follows:

(4) $$J_{w}=\frac{Q}{A\Delta t}$$

where *V* (L) is the volume of permeate water, *A* (m^2^) is the working membrane area, and Δ*t (h)* is the separation time.

The antifouling performance of membranes and the flux recovery ratio (FRR) were calculated using the following equation:

(5) $$\mathrm{FRR}=\frac{J_{c}}{J_{0}}\times 100$$

where *J*_0_ is the flux of pure water (L·m^2^ h^−1^) before filtration of the BSA solution and *J_c_* is the flux of pure water after rinsing the used membrane (L·m^2^ h^−1^).

## 3. Results and Discussion

### 3.1. Membrane Characterization

#### 3.1.1. FTIR of Neat and Incorporated CA Membranes

**Figure 1:**
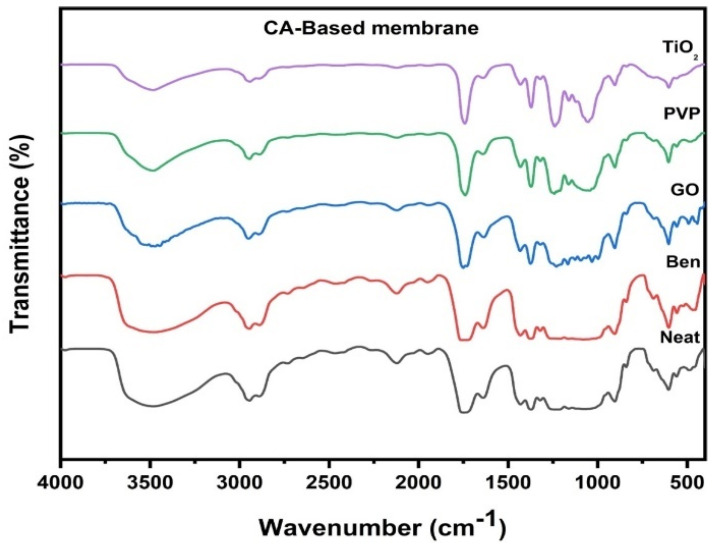
FTIR of CA-based composite membranes.

Figure 1 demonstrates the FTIR spectra of the neat and integrated CA membranes made of PVP, B, TiO_2_, and GO. The strong –OH groups extending to the orderly CA membrane were attributed to the 3480.3 cm^−1^ peaks [45]. Furthermore, the characteristics of CA membranes like C–H stretching, strong C=O stretching, and C–O stretching were each ascribed to the peaks at 2944.34 cm^−1^, 1745.3 cm^−1^, and 1121.01 cm^−1^, respectively. The peaks in the CA-Ben membrane at 3477.96 cm^−1^ and 2731.84 cm^−1^ were attributable to significant –OH stretching in carboxylic acid groups [43]. Additionally, 2121.56 cm^−1^, 1214.61 cm^−1^ and 687.9 cm^−1^ were assigned to the C≡C stretching, strong C-O stretching, and strong C=C bending alkene compounds, respectively. In the CA-GO membrane, the peak at 3516.41 cm^−1^ corresponds to -OH stretching in the alcohol group [46]. Additionally, 2949.63 cm^−1^ peaks were allocated to -OH stretching in the carboxylic acid group and the peaks at 1750.89 cm^−1^, 1432.69 cm^−1^, and 1125.38 cm^−1^ were attributed to the -CH bending aromatic compound group, -OH bending carboxylic acid compound, and S=O stretching, respectively. The peak at 684.84 cm^−1^ was assigned to strong C=C bending, and in the CA-PVP membrane, the peak at 3484.56 cm^−1^ was allocated to the strong O-H stretching alcohol group and 2944.50 cm^−1^ to the strong N-H stretching in the amine salt compound. Furthermore, the peaks at 2890.3 cm^−1^, 1948.89 cm^−1^, and 1640.65 cm^−1^ were consigned to the –OH stretching in the alcohol compound, the -CH bending in the aromatic compound, and strong C=C stretching, respectively [47]. The 1370.71 cm^−1^ and 1042.67 cm^−1^ peaks correspond to strongly stretched N-O in the nitro compound and strong CO–O–CO stretching in the anhydride molecule, respectively. In the CA-TiO_2_ membrane, peaks at 3483.04 cm^−1^ and 2942.40 cm^−1^ were assigned to the strong –OH stretching of the alcohol compound and strong –OH stretching of the carboxylic compound, respectively. Additionally, peaks at 2120.80 cm^−1^, 1741.81 cm^−1^, and 1239.33 cm^−1^ were apportioned to C≡C stretching, strong C=O stretching, and strong C–O stretching, respectively. Further, the peaks at 1052.00 cm^−1^ and 838.30 cm^−1^ were attributed to the strong C–O stretching of the primary alcohol compound and C=C bending of the alkene compound. Interestingly, in the membrane matrix with the nanomaterials bentonite, GO, PVP, and TiO_2_, the –OH group on the membrane surface was identified to enhance hydrophilicity and oil removal in water [37].

#### 3.1.2. Morphological Studies of CA-Incorporated Membranes

**Figure 2:**
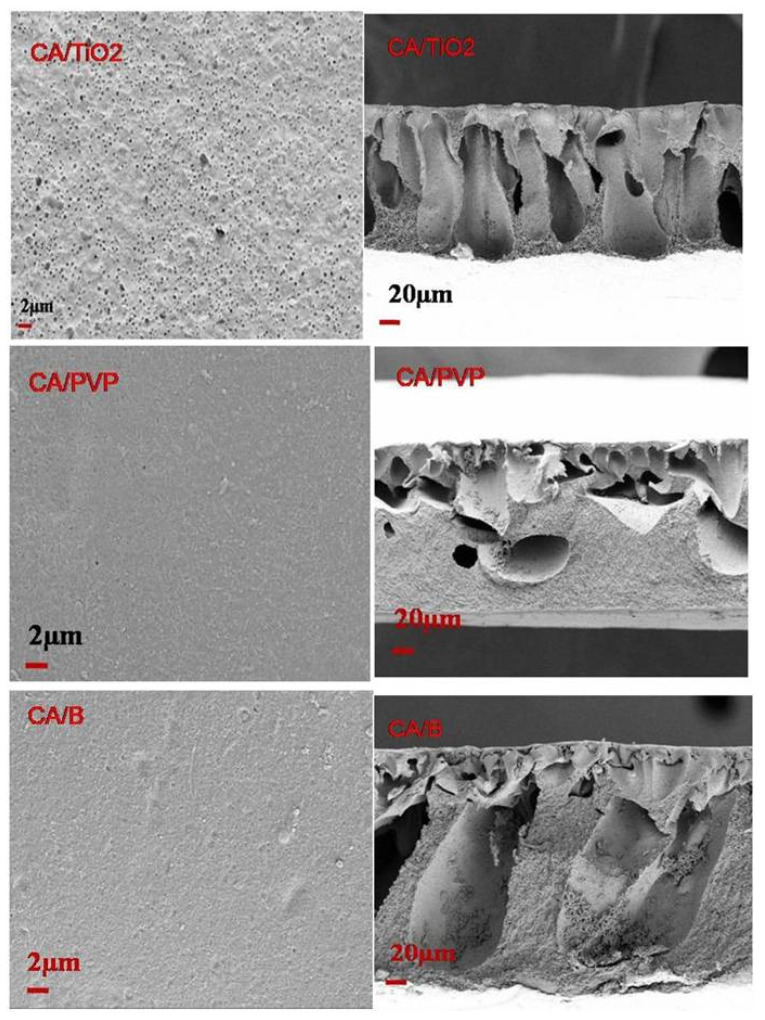
Top and cross-sectional morphology of the CA-based composite membranes.

The top and cross-sectional images of CA-TiO_2_, CA-PVP, and CA–bentonite are displayed in Figure 2. The presence of nanomaterials in the top surface morphology is represented as white particles and the dense skin layer is obviously clear. In the fabricated CA-TiO_2_, CA-PVP, and CA-B membranes, the asymmetric structure was assumed to be a thick skin layer with a porous structure. Nanomaterials are considered to accelerate the phase separation process and affect the thermodynamic permanence of the casting solution when added to polymers, and the nanomaterials and polymer matrix membranes [48,49,50,51] have higher compatibility, which may be provided by the nanomaterials isolated uniformly with the polymers. Macrovoid structures were recognized in the porous sublayer of the CA-incorporated membrane cross-section and were composed of finger-like structures; they have been reported to reduce water transport resistance and enhance water flux in the membrane phase separation process [52].

It was also observed from the cross-sectional SEM images that the high amounts of TiO_2_ nanoparticles made the layer very dense, and the finger-like structures appeared to be slightly more established. Compared with the CA-B solution, the inclusion of CA-TiO_2_ nanoparticles in the casting solution greatly accelerated the exchange between the solvent and non-solvent, resulting in finger-like microvoids and greater porosity [53,54]. The incorporation of nanoparticles into the CA surface membrane enhanced its effectiveness in removing oil from the emulsion. These nanoparticles might have specific properties that improve the adsorption of oil droplets and help further stabilize the emulsion. The CA, combined with nanoparticles such as graphene oxide, bentonite, or titanium dioxide, aided in the reduction of the interfacial tension between oil and water, stabilizing the emulsion. The incorporation of nanoparticles into the CA matrix probably enhanced the oil adsorption capacity of the membrane. On the other hand, as the amount of TiO_2_ nanoparticles increased, agglomerates of the nanoparticles were observed. The incorporated CA-TiO_2_ membrane thereby heightened the transport properties and enhanced the hydrophilic finger-like microvoid groups in the sublayer [39].

**Figure 3:**
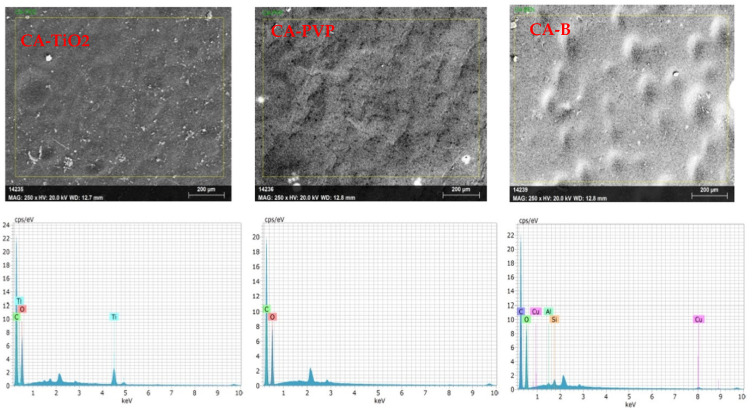
EDAX images for identifying the elemental composition of TiO_2_, PVP, and bentonite materials in CA membranes.

Figure 3 represents the Energy-Dispersive X-ray Spectroscopy (EDAX) spectra of CA-TiO_2_, CA-PVP, and CA-Ben. EDAX is a valuable analytical technique that is used primarily for elemental analysis and can provide important information regarding the composition of materials. For oil/water emulsion separation or removal of oil, EDAX was used to verify and confirm the presence of specific elements or materials in the membranes used in this study. The EDAX spectrum of the CA-TiO_2_-incorporated membrane displayed peaks corresponding to carbon, oxygen, and titanium. No other elemental peaks were detected, confirming the presence of oxygen or Ti. The figure also reveals the EDAX spectrum of the CA-PVP membrane in which carbon and oxygen peaks can be observed. The absence of other elemental peaks suggested that the CA-PVP membrane was primarily composed of carbon and oxygen, thereby justifying the presence of polyvinyl pyrrolidone (PVP). Likewise, the figure corroborated the presence of carbon, oxygen, aluminum, and silicon, confirming that the bentonite clay materials were found in CA–bentonite membrane blends [55]. The SEM results confirmed that the intended materials or additives (e.g., TiO_2_, PVP, and bentonite) were accurately incorporated into the membranes, and the results also provided valuable information about the presence of specific materials, which was crucial for ensuring the effectiveness of the separation process.

#### 3.1.3. Contact Angle and Swelling Degree for Membranes

**Figure 4:**
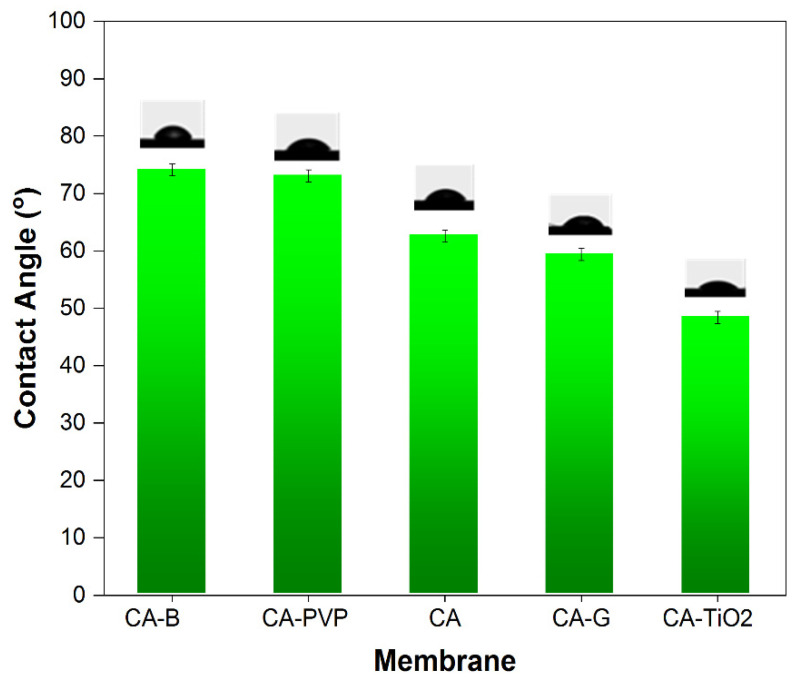
Water contact angles of CA-based composite membranes.

All the incorporated membranes were cleaned and dried in a vacuum oven for 24 h at 100 °C before applying them in the contact angle experiment [56]. The water contact angle (CA) values (Figure 4) provided insights into the wettability of the membrane surfaces. A smaller contact angle indicated better wettability, suggesting that water spreads more easily across the surface. The water contact angle of the neat CA membrane is 62.6°. The contact angle values increased in CA-Ben and CA-PVP and decreased in CA-GO and CA-TiO_2_ with the inclusion of CA nanomaterials: 74.1°, 71.3°, 59.4°, and 48.4°, respectively. These results thus indicated that the low contact angles of the membrane surface hydrophilicity were enlarged. The incorporated CA membranes exhibited increased hydrophilicity and enhanced hydrogen bonding with water-containing compounds. From the perspective of oil removal from oil/water emulsions, a lower contact angle is generally more favorable as it indicates that the membrane is more hydrophilic and, therefore, has a better affinity for water. This enhanced wettability facilitates the separation of oil from the emulsion [57]. In comparison to the neat CA membrane, the contact angles of the CA-TiO_2_ and GO membranes decreased, which implies the presence of a greater number of hydroxyl groups in both TiO_2_ and GO compared to PVP and bentonite. The addition of hydrophilic nanomaterials of TiO_2_ and GO to the CA mixed-matrix membranes resulted in higher wettability. These two membranes (CA-GO and CA-TiO_2_) were the most suitable for the removal of oil from oil/water emulsions because of their better wettability. Their hydrophilic nature indicates that they are likely to interact well with the water phase in the emulsion, which is beneficial for efficient oil separation [58].

**Figure 5:**
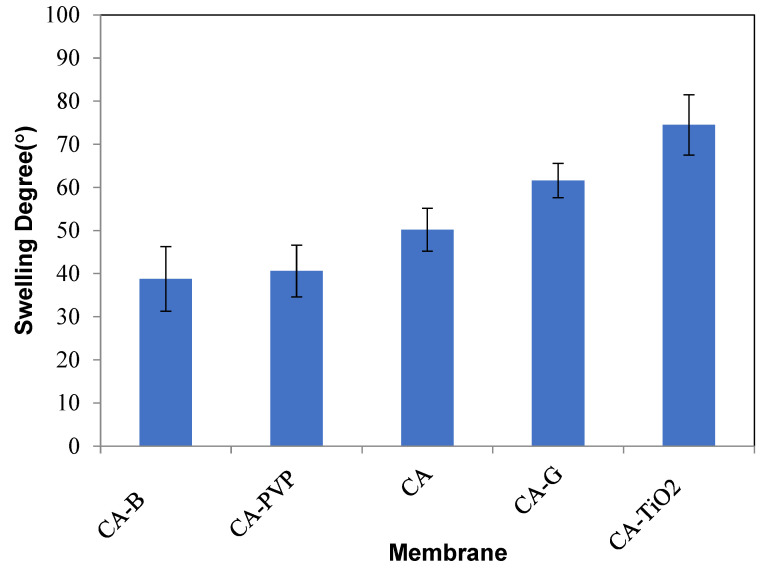
Swelling degree of CA-based composite membranes.

Figure 5 displays the swelling degrees of neat CA, CA-B, CA-PVP, CA-GO, and CA-TiO_2_-incorporated membranes. The swelling degree of membranes is a critical factor in their performance for the removal of oil from oil/water emulsions. This affects the ability of the membrane to absorb or interact with the emulsion components, and the choice of swelling degree is dependent on the specific requirements of the separation process [59]. The swelling degrees of these membranes can be interpreted from the perspective of oil removal from oil/water emulsions based on the forthcoming facts. A high swelling degree indicated the fact that the CA-TiO_2_ membrane was capable of absorbing a significant amount of liquid, which could be advantageous for oil removal, whereas the swelling degree of CA-TiO_2_ composite membranes was observed to be higher than that of other membranes due to hydrophilicity and low contact angles. The extensive swelling condition specified that it absorbed more emulsion, thus potentially improving the separation efficiency [60]. In accordance with the justification mentioned, the CA-GO membrane also showcased a relatively high swelling degree, suggesting good absorbency and potential for effective oil removal, whereas neat CA exhibited a moderate swelling degree. They possessed a certain capacity to absorb emulsion, which rendered them suitable for general oil removal applications. CA-PVP revealed a lower degree of swelling than neat CA, which designated a lower capacity for emulsion absorption. However, this could be advantageous if the goal was to minimize swelling and retain the integrity of the membrane during oil/water separation. CA–bentonite had the lowest swelling degree, indicating limited capacity for liquid absorption. This could be beneficial in applications where maintaining the structural integrity of the membrane is considered a top priority. The cross-linked polymers of the nanoclay and CA-PVP-incorporated membranes exhibited a lower degree of swelling.

#### 3.1.4. Pure Water Flux of Membranes

**Figure 6:**
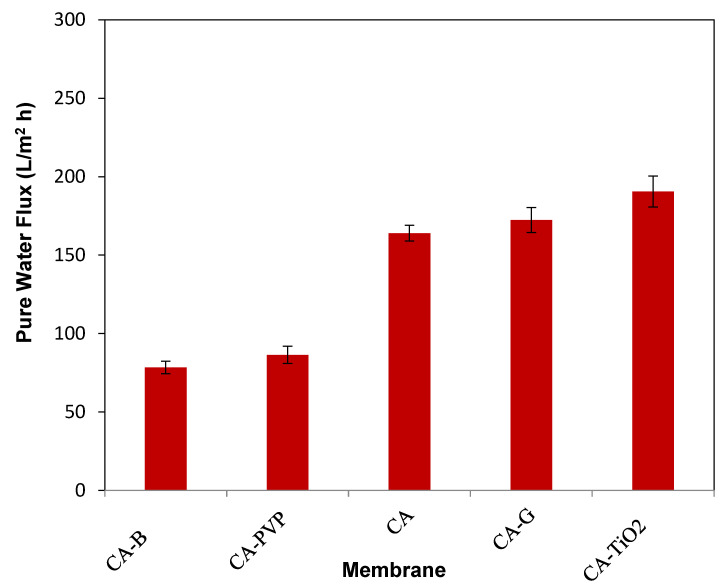
Pure water flux (PWF) of CA-based composite membranes.

The pure water flux (PWF) of the membranes was calculated at different intervals at 30 min, 345 kPa transmembrane pressure (TMP), and 30 °C, as observed in Figure 6. The pure water flux values of CA-TiO_2_ were higher than those of the other membranes because of their low contact angles. The pure water flux values of CA-TiO_2_ were calculated and were noticed to be higher than those of neat CA and other composite incorporated membranes of CA in different mixtures of oil/water emulsions. Similarly, the addition of the TiO_2_ nanoparticles into the CA membranes resulted in the enhancement of the hydrophilic properties of the membrane since it had a greater number of hydroxyl groups, which in turn resulted in a reduction in the contact angle. The hydroxyl groups’ prevalence on the surface of the nanoparticles enhanced the interaction with water molecules, which resulted in a greater pure water flux ability than the CA membrane. Correspondingly, the CA-GO membrane achieved superior pure water flux to the CA membrane but was not much more significant than CA-TiO_2_. This was also due to the lower contact angle of CA-GO, which was due to the presence of a greater number of hydroxyl groups. In our previous study, we reported that the TiO_2_ nanomaterials have catalytic property and responsible for enhanced oil/water removal efficiency and high flux property [61]. In addition, the hydrophilic groups present in the TiO_2_ and GO nanoparticles accelerated rapid de-mixing and caused an increase in thermodynamic instability, which increased the porosity of the skin layer in the nanoparticle-embedded CA membranes. This resulted in an overall augmentation in the pure water flux of the nanoparticle-embedded CA membranes.

#### 3.1.5. Flux Recovery Ratio (FRR)

**Figure 7:**
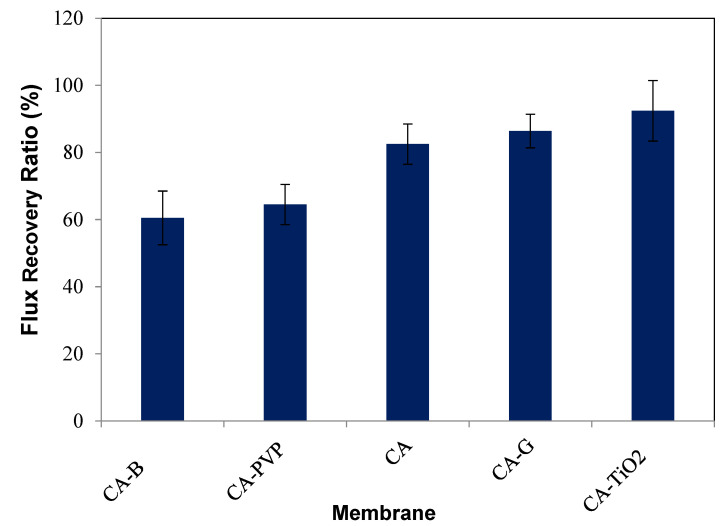
FRR for CA-based composite membranes.

Figure 7 represents the flux recovery ratios of the membranes. The flux recovery ratio (FRR) is the measure of the conversion ratio after the water was supplied through the membrane separation (solution). The flux recovery ratio was calculated as per Equation (5) [62]. The FRR studies were performed using an ultrafiltration setup, with a volume of 400 mL and a membrane effective area of 38.5 cm^2^. Clean water and a synthetic oil/water mixture were used for flux studies prior to and after oil/water mixture separation at 345 kPa TMP. The flux recovery ratio of CA-TiO_2_ was higher than that of the CA, CA-B- and CA-PVP-incorporated membranes. It has been considered that a high FRR represents an enhancement of the antifouling property, and with the incorporation of TiO_2_ and GO into CA membranes, FRR was evaluated as 95.6% and 92.5%, respectively. Therefore, the CA-TiO_2_ membrane had confirmed positive effects on the performance and also revealed a considerable superiority in separation efficiency and flux recovery. CA-G also had a relatively high FRR value, indicating good recovery after cleaning, although neat CA with a moderate FRR value showed a reasonable ability to recover its filtration capacity. CA-PVP exhibited a lower FRR compared to pure CA, indicating a reduced ability to recover its initial filtration capacity after cleaning. CA-B, with the lowest FRR among the options, indicated that it may have more difficulty in regaining its filtration capacity after cleaning. The CA-TiO_2_ composite membrane demonstrated a heightened separation performance for oil solutions, with an excellent flux recovery ratio under pressure in UF membranes. A higher FRR value of CA-TiO_2_ demonstrated its better ability to regain and maintain its filtration performance after fouling or clogging, which is essential in processes such as the removal of oil from oil/water emulsions.

## 4. Oil-in-Water Emulsions on Filtration Studies

### 4.1. Rejection of Oil from CA Neat and Composite Membranes of Permeate Flux

**Figure 8:**
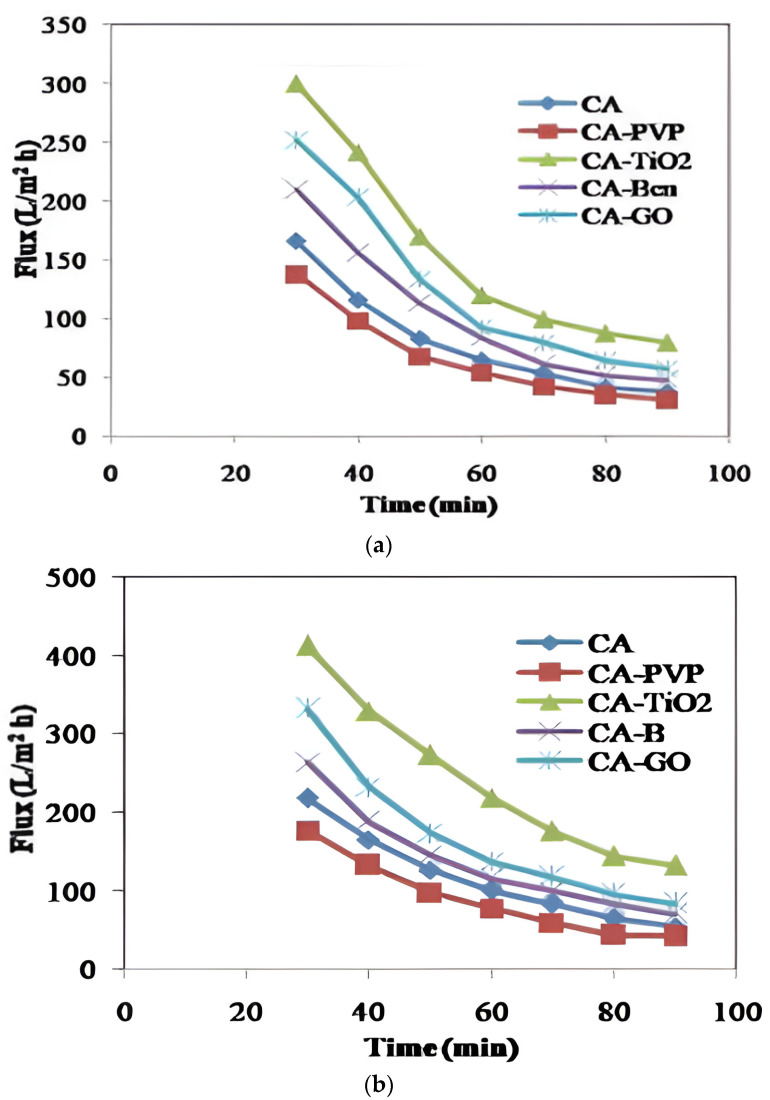

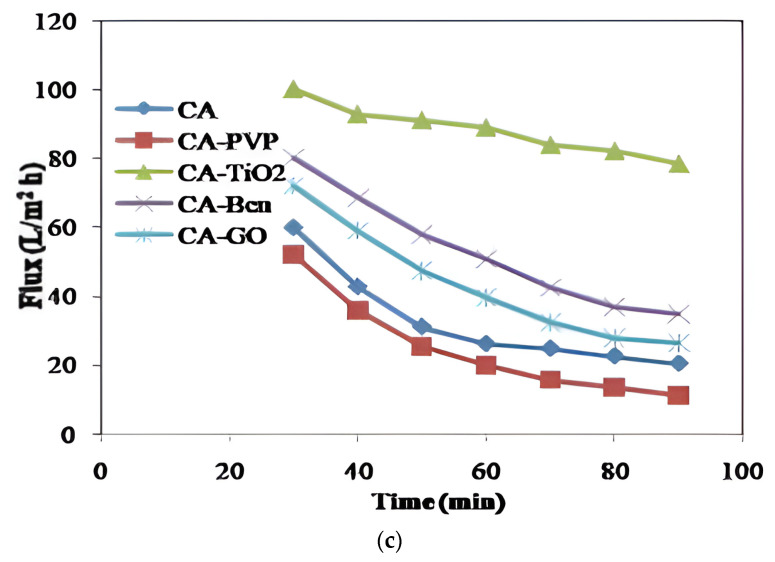
Permeate flux of oil/water emulsions with time. (**a**) Permeate flux of hexane/water emulsion. (**b**) Permeate flux of toluene/water emulsion. (**c**) Permeate flux of engine oil/water emulsion.

From the perspective of oil removal from oil/water emulsions, a higher flux value is generally indicative of a membrane’s ability to process a larger volume of the emulsion per unit of time. The performance of each manufactured membrane was evaluated using oil/water combinations. After measuring the pure water flux at 345 kPa, the feed was transformed into an oil/water mixture (pH 7). The oil/water mixture was filtered for 180 min at 30 °C and 345 kPa TMP. Figure 8 exhibits the membrane permeate flux with hexane/water, toluene/water, and engine oil/water mixtures at various time intervals. The flux values of CA-TiO_2_ were observed to be higher than those of the neat and other CA-incorporated membranes of hexane/water, toluene/water, and engine oil/water mixtures concerning different time intervals. The flux values of the CA-incorporated membranes in hexane/water, toluene/water, and engine oil/water mixtures attained a decreased value and fascinatingly, CA-TiO_2_ consistently exhibited higher flux values. This outcome validated that this membrane encompassed a better permeability and was more efficient at allowing the passage of water or the separated phase (likely water) through the membrane while retaining the oil phase [63]. This higher flux was remarkable as it determined that the membrane was capable of processing larger volumes of oil/water emulsion in a given time, making it more efficient for oil removal [63]. Therefore, the flux values of the CA-TiO_2_ membrane were consistently higher than those of CA-GO, CA-PVP, CA, and CA–bentonite at different time intervals. The CA-TiO_2_ membrane was noteworthy for the removal of oil from oil/ water emulsions in hexane/water, toluene/water, and engine oil/water mixtures. CA-GO, CA-PVP, CA, and CA–bentonite membranes consistently illustrated lower flux values than CA-TiO_2_, which suggested that they may have reduced permeability and lower efficiency in terms of processing the emulsion. These results indicated the fact that they process smaller volumes of emulsion per unit of time or may be easily fouled, thereby reducing their overall effectiveness in oil removal. Higher flux values indicated better efficiency, advanced throughput, and a more effective separation process, which were highly crucial factors in oil/water emulsion separation applications.

### 4.2. Rejection Studies on Oil-in-Water Emulsion Filtration by CA-Incorporated Membrane

#### 4.2.1. Oil/Water Emulsion Filtration of Composite Membranes of CA in Toluene/Water Mixture

**Figure 9:**
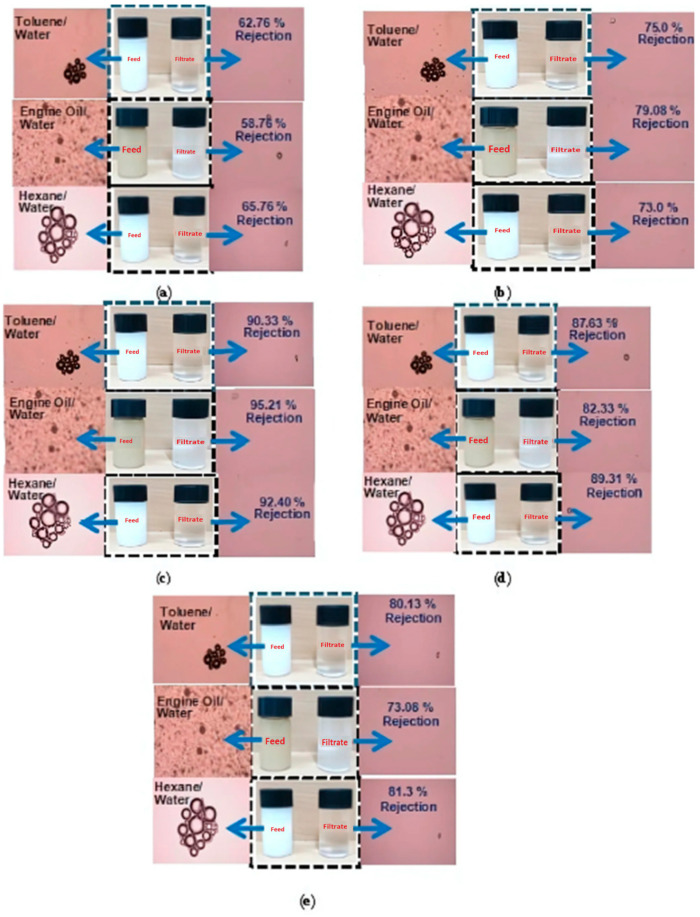
Rejection of oil/water emulsion before and after filtration. (**a**) CA membrane. (**b**) CA-PVP membrane. (**c**) CA-TiO_2_ membrane. (**d**) CA-B membrane. (**e**) CA-GO membrane.

In a toluene/water mixture, the filtering of CA and the incorporated composite membrane of the oil/water emulsion are demonstrated (Figure 9a–e). A mixture of emulsions prior to and post filtration of neat CA and membranes incorporating PVP, TiO_2_, bentonite and GO were found in toluene/water mixtures. In neat CA and CA-PVP, CA-TiO_2_, CA-B, and CA-GO, oil rejection values were 62.76%, 75.0%, 90.33%, 87.63%, and 80.13%, respectively, in toluene/water mixtures. In CA-TiO_2_-incorporated membranes, the oil rejection was higher than that of the neat and other membranes in toluene/water mixtures. The antifouling performance of the CA membrane was superior, and thereby the oil rejection values were reduced in engine oil/water after the ultrafiltration membranes were used [64].

#### 4.2.2. Oil/Water Emulsion of Incorporated Membranes of CA in Engine Oil/Water Mixture

Figure 9a–e display the representation of the oil/water emulsion for CA and the incorporated composite membrane in the engine oil/water mixture. In engine oil/water mixtures, emulsions of CA, CA-PVP, CA-TiO_2_, CA-B, and CA-GO subsisted both before and after filtration. Employing a microscope for visual clarity assessment after ultrafiltration has been considered a practical approach to qualitatively evaluate the effectiveness of oil removal from engine oil/water mixtures [65]. A microscope facilitates visual assessment of the permeate clarity (filtrate) obtained after ultrafiltration, and a clear permeate indicates that a significant amount of oil has been successfully removed from the mixture. A less turbid permeate suggests that the membrane has effectively separated the oil phase from the water phase [62,66]. The oil rejection was higher in CA-TiO_2_ and CA-B due to the hydrophilicity of the membrane and low contact angles in the engine oil/water mixtures. In the incorporated membranes, the CA, CA-PVP, CA-TiO_2_, CA-B, and CA-GO oil rejection values were 58.76%, 79.08%, 95.21%, 82.333%, and 73.08%, respectively. Microscopes can also help in observing the size and nature of any remaining particles or contaminants in the permeate. Smaller particles or droplets indicate that the membrane has effectively removed larger oil droplets. Using a microscope to compare the clarity of the permeate from the different membranes (CA, CA-PVP, CA-TiO_2_, CA-B, and CA-GO), the membrane that produces the clearest permeate is likely to be the most effective for oil removal. In membranes incorporating CA-TiO_2_, the engine oil/water mixture rejection was greater than that in membranes incorporating other incorporated materials.

#### 4.2.3. Oil/Water Emulsion of Incorporated Membranes of CA in Hexane/Water Mixture

Figure 9a–e depict the visual representation of emulsion filtration for pure CA, and the hexane/water mixture represents the incorporated composite membrane. The results have clearly indicated that the hexane/water mixtures contained emulsions of CA, CA-PVP, CA-TiO_2_, CA-B, and CA-GO before and after filtration. In hexane/water mixtures, oil rejection values in CA, CA-PVP, CA-TiO_2_, CA-B, and CA-GO membranes were 65.76%, 73.00%, 92.04%, 95.02%, 89.33%, and 81.3%, respectively. In oil/water emulsions with hexane/water compositions, the oil rejection of membranes incorporating CA-TiO_2_ was greater than that of membranes incorporating other materials. The CA-TiO_2_-incorporated membrane employed for the filtration of oil in toluene, hexane, and engine oil mixtures with water had oil rejection values 90.33%, 92.4%, and 95.21% higher than those of neat CA and the other incorporated membranes. The increased attraction between the membrane surface and oil droplets might have been caused by the surface hydrophilicity, reduced interface, and incorporation of TiO_2_, resulting in a higher rejection of oil [66,67]. In the automobile industry, oils such as engine oils or tractor fluids have an even greater affinity for moisture. The emulsion is a cloudy oil/water mixture that is tightly bound with a little-to-no-separation tendency. From the obtained experimental outcomes, it was evident that a small amount of TiO_2_ in membranes efficiently resulted in 95% oil/water separation efficiency and effectively proved to be significant for the automobile industry to treat tightly bound oil/water mixtures due to the excellent wettability and pore size reduction of TiO_2_ nanoparticles.

## 5. Conclusions

This research investigation is a detailed description of oil/water emulsion wastewater treatment using incorporated (CA-TiO_2_, CA-B, CA-PVP, and CA-GO) membranes. Nanomaterials such as TiO_2_, GO, the nanoclay material bentonite, and PVP were incorporated into CA membranes and were fabricated by a casting solution using a phase inversion technique with NMP as a solvent. The phase inversion (PI) technique is an important strategy to integrate nanomaterials into polymeric membranes. Hydrophilic additives (PVP) and nanoparticles were immensely capable of accelerating the exchange of organic solvent and non-solvent (water) in the PI process. Water diffusion into the thin polymer films resulted in a potential enhancement in the membrane porosity. The nanomaterial-incorporated membranes exhibited better hydrophilicity, porosity, and pore size than those of the neat CA membrane. FTIR studies exposed the existence of the -OH group, which might have played a crucial role in enhancing the hydrophilicity and oil removal of the mixed-matrix membrane surfaces (bentonite, GO, PVP, and TiO_2_). The -OH groups and hydrophilicity boosted the pure water flux values in the CA-TiO_2_ membranes. The CA-TiO_2_-incorporated membranes with a water contact angle of 48.5° demonstrated better hydrophilicity than the CA, CA-GO, CA-B, and CA-PVP composite membranes. The hydrophilic groups were responsible for the reduction in the spongy layer, as observed in the cross-sectional morphology of the CA-TiO_2_-incorporated membranes. The addition of nanomaterials also affected the fouling behavior of the membranes. According to the FRR data, TiO_2_ nanomaterials lead to the improvement of membrane hydrophobicity, which is more susceptible to oil/water fouling and results in higher fouling resistance. Several different oil/water mixtures were separated using membranes, and the separation efficiency reached 95% of the engine oil/water mixtures for the CA-B membranes. In summary, this research endeavor is a potential attempt to effectively highlight the applications of CA-TiO_2_, CA-B, CA-PVP, and CA-GO membrane materials in the field of oil/water separation.

## Data Availability

The data presented in this work are available upon request from the corresponding authors.
